# India’s journey in mainstreaming Ayush in primary health care—from tradition to integration

**DOI:** 10.3389/fmed.2025.1629515

**Published:** 2025-10-28

**Authors:** Tanuja Nesari, Manoj Nesari, Galib Ruknuddin, R. K. Yadava, Vittal Huddar, Prasanth Dharmarajan, Shivani Ghildiyal, Prashant Kumar Gupta, Ramesh Kumar, Varun Gupta, Deep Shikha Punera, Shifa Shetty P, Sumeet Goel

**Affiliations:** ^1^Institute of Teaching and Research in Ayurveda, Jamnagar, India; ^2^Central Government Health Scheme, Ministry of Health and Family Welfare, Government of India, New Delhi, India; ^3^All India Institute of Ayurveda, New Delhi, India; ^4^Centre of Excellence for Applied Development of Ayurveda Prakriti and Genomics, CSIR Institute of Genomics and Integrative Biology, New Delhi, India; ^5^All India Institute of Ayurveda, Goa, India

**Keywords:** traditional medicine, Ayush systems, primary health care (PHC), integrative healthcare, universal health coverage (UHC), evidence-based Ayurveda, National Ayush Mission, health policy and systems integration

## Abstract

**Introduction:**

India has systematically integrated Indian traditional medicine systems Ayurveda, Yoga, Unani, Siddha, Sowa-rigpa including Homeopathy —collectively known as ‘Ayush’ into its public healthcare delivery. Since upgrading the Department of Ayush to a dedicated Ministry of Ayush in the year 2014, several landmark initiatives have been launched, including the National Ayush Mission (NAM), the establishment of Ayush Health and Wellness Centres under Ayushman Bharat, and the creation of the Ayushman Arogya Mandirs network. These efforts reflect a strategic commitment to enhance primary health care (PHC) by promoting culturally relevant, preventive, and affordable services.

**Methods:**

This practice and policy review employed a systematic approach to analyze the integration of Ayush into India’s PHC system. Primary and secondary source of data was drawn from national health policies, government reports, international frameworks, and official statistics between 2014 and 2024. Data was analyzed in detail to assess implementation status, infrastructure, global positioning, education, digital integration, and policy challenges.

**Observations:**

India’s traditional medicine sector includes 12,500 Ayushman Arogya Mandir led by qualified doctors of Ayurveda, Unani, Siddha, Sowarigpa and homeopathy doctors, 750,000 registered institutionally qualified practitioners, more than 700 Ayush medical colleges and attached hospitals, around 9,000 Ayush drug manufacturing industries, dedicated research councils for ewach of the Ayush system with their peripheral centers, a Pharmacopoeia Commission of Indian systems of Medicine and Homeopathy etc. Moreover, Ayush systems are integrated in 26,636 Primary Health Centres (PHCs), 6,155 Community Health Centres (CHCs), and 759 Districts Hospitals (DH) in the country. Ayush systems are also integrated in health infrastructure under Ministry of Defense, Ministry of Labour Welfare, Ministry of Railways etc. Public health programs targeting maternal health, geriatric care, and non-communicable diseases have incorporated Ayush-based approaches. Internationally, India has established academic collaborations and information cells across 42 countries and academic chairs across 38 countries, while domestic initiatives focus on digital health (Ayush Grid), education reform (NEP 2020), quality assurance, and cross-referral pathways.

**Inference:**

India’s integrative approach demonstrates how traditional medicine can enhance PHC delivery, particularly in underserved settings. With continued investment in evidence-based practices, regulatory alignment, and inclusive models, Ayush can play a pivotal role in achieving Universal Health Coverage and informing global traditional medicine strategies.

## 1 Introduction

India’s traditional medicine systems—Ayurveda, Yoga and Naturopathy, Unani, Siddha and Sowa rigpa have been an integral part of primary healthcare in households of Indians for centuries while Homeopathy is often considered alongside Traditional Indian system of Medicine and has been integrated and adopted into the country’s healthcare landscape for over two centuries—all of which have been regulated under the ambit of Ministry of Ayush. Each of these systems brings distinct epistemological frameworks and therapeutic approaches, collectively contributing to the landscape of primary health care (PHC) in the country. While Ayurveda has historically dominated scholarly and clinical attention, other Ayush systems are also accepted by the community, particularly in certain geographic regions Under the leadership of the Ministry of Ayush, the National Ayush Mission is taking this legacy forward by establishing Ayush health and wellness centers, co-locating Ayush services in existing health facilities, and supporting infrastructure, manpower, and medicine supply to ensure holistic and accessible healthcare.

In India formal education in traditional medicine evolved over thousands of years shifting from the Gurukul system (mentor-mentee training system, where knowledge was passed down orally or via apprenticeships within families) (1) to world class ancient Universities, e.g., Nalanda, Taksheela, Vikram Sheela in 3rd century BC to institutionalized university schooling system starting in the early 20th century. In the 20th century, the establishment of the first Ayurveda college in 1908, followed by the creation of regulatory bodies which are formal and legalized by the Act of parliament to bring uniformity in Ayush education system through NCISM and NCH (formerly known as CCIM and CCH).

Simultaneously, research ecosystem is fostered by establishing research councils such as CCRAS, (Central Council for Research in Ayurvedic Sciences), CCRH (Central council for Research in Homeopathy), CCRUM (Central council for Research in Unani Medicine), CCRS (Central council for Research in Siddha), and CCRYN (Central council for Research in Yoga and Naturopathy), and launched digital knowledge platforms like the Ayush Research Portal (2). Along with the extensive number of studies available, it was observed that corrective measures are being taken by the councils to upgrade research methodologies and their outcomes as per international standards.

In recent years, the Government of India has strategically boosted up efforts of harnessing the potential of Ayush systems into mainstream primary healthcare activities including creating separate Ayush infrastructure, optimizing Ayush trained HR and ensuring availability of therapies and medicines at grassroot level integrating with PHC network to contribute to prevention, health promotion, cultural relevance, and low-cost interventions, especially in underserved regions. This integration process involves both opportunities and challenges, including validation through scientific research, the development of collaborative models with biomedicine, and concerns about quality assurance and equitable access.

Globally, the World Health Organization (WHO) recognizes Traditional, Complementary, and Integrative Medicine (TCIM) as experience-based health systems that can be aligned with national healthcare frameworks when implemented safely and evidence-based. India’s Ayush integration efforts align with WHO’s Traditional Medicine Strategy 2025–2034, which advocates for rigorous evidence, quality standards, and equitable implementation (3).

**Figure 1:**
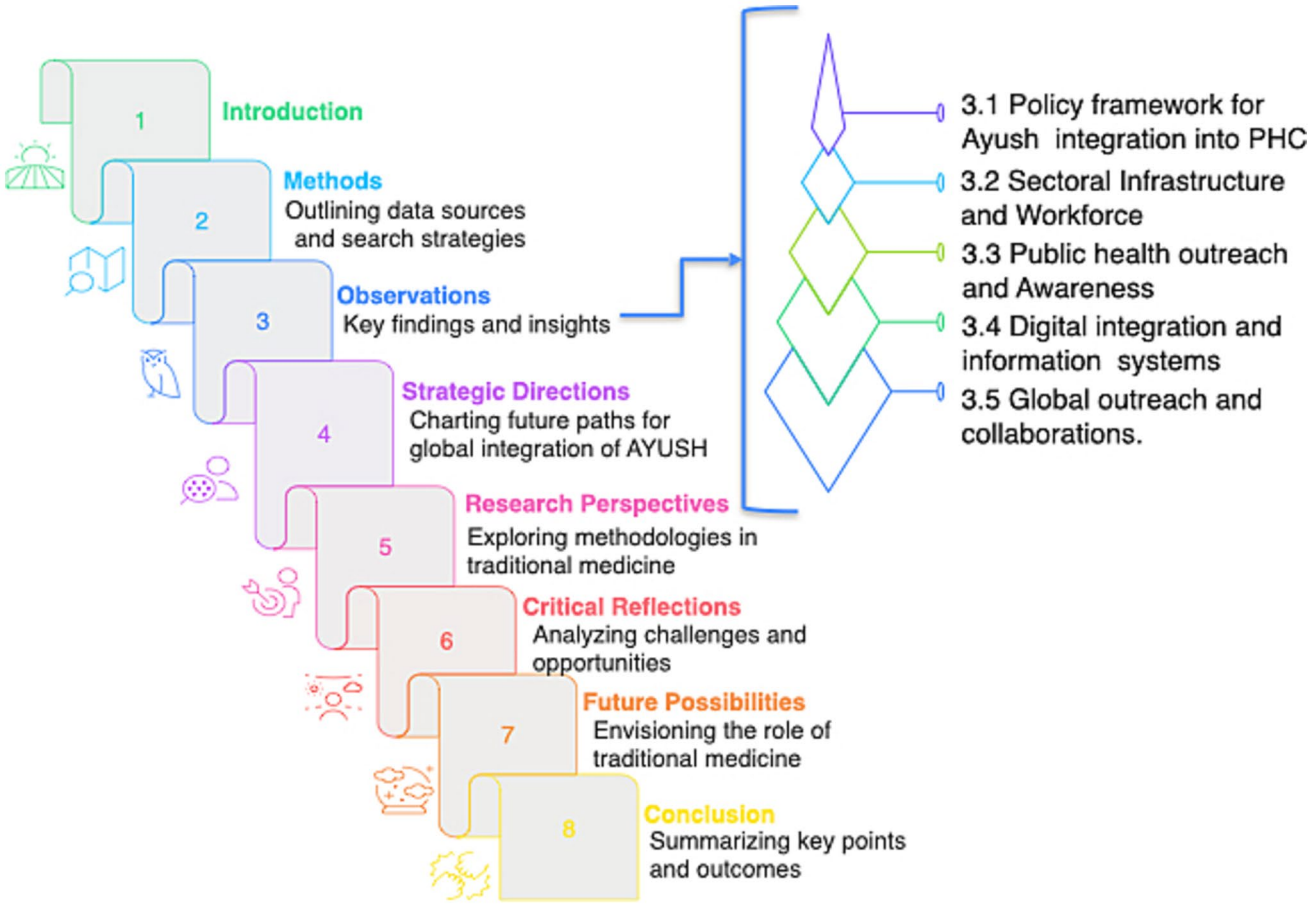
Infographic illustrating the overall structure and flow of this review, outlining the key sections and concepts discussed throughout the manuscript.

To collate all the efforts of Ayush system towards Primary Health Care as a recognized healthcare system. This review was envisioned to analyze the implementation, scope, and outcomes of Ayush integration into India’s PHC from 2014 to 2024. It aims to evaluate both achievements and gaps by synthesizing data from national health programs, international collaborations, institutional developments, and public health indicators relevant to traditional medicine. An infographic summarizing the overall concept and flow of this manuscript is presented in Figure 1.

## 2 Methods

This review adopted a systematic, mixed-methods approach to examine the integration of Ayush systems into India’s primary health care (PHC) framework from 2014 to 2024. The study was collaboratively conducted by a multi-disciplinary team comprising researchers, Ayush clinicians, and policy analysts.

We employed a policy and program analysis framework based on the WHO’s health system building blocks (service delivery, health workforce, information systems, access to medicines, financing, and governance). The team conducted an iterative search and synthesis of national and international policy documents, government program reports, and academic literature.

### 2.1 Data sources and search strategy

Relevant data were sourced from the following platforms:

- Government repositories: Ministry of Ayush website, Press Information Bureau (PIB), and Ministry of Statistics and Programme Implementation (MOSPI), National Health Policy 2017
- International databases: WHO IRIS and WHO Traditional Medicine publications
- Academic databases: Google Scholar and PubMed
- Inclusion Criteria:
  - Timeframe: 2014 to 2024
  - Type of Sources: Peer-reviewed publications, national and international policy documents, official Ayush statistics, and validated institutional reports
  - Scope: Ayush integration in healthcare systems, education, globalization, and digital infrastructure
  - Language: English only

### 2.2 Review process

Each source was independently screened by at least two reviewers for relevance and quality. Discrepancies were resolved through discussion and consensus. Thematic coding was conducted using manual methods to group information under key domains such as policy implementation, service delivery models, education and regulation, public health outreach, digital innovations, and global engagement.

A data triangulation strategy was employed to validate findings across multiple sources. In cases where quantitative data were ambiguous or inconsistent, corroboration was sought from alternate repositories or direct institutional reports. Limitations in data availability or consistency are noted. The details of the sources reviewed are given in Table 1.

**Table 1:** Tabulated summary of key sources consulted.

Source/Platform	Type of document	Access URL	Focus/Use in review
Ministry of Ayush Annual Reports (2014–2024)	Government reports	http://www.dbtayush.gov.in/resources/pdf/annualReport/AR_2024_2025.pdf	Programmatic coverage, service delivery models
Ayush Decade Growth Report (2014–2024)	Decadal summary report	https://aiia.gov.in/wp-content/uploads/2024/03/AyushDecadeGrowthReport-Eng-Final.pdf	Sector-wide achievements, schemes, reforms
WHO Traditional Medicine Strategy 2014–2023	Global policy document	https://www.who.int/publications/i/item/9789241506096	Global integration strategy, UHC alignment
WHO SEARO Traditional Medicine Progress	Regional review report	https://iris.who.int/handle/10665/340393	South-East Asia regional progress 2014–2019
NSSO 79th Round Report (2022–2023)	National Health Survey	https://www.mospi.gov.in	Ayush utilization, health-seeking behavior
Press Information Bureau (PIB)	Press releases	https://www.pib.gov.in/PressReleasePage.aspx?PRID=1740732	Trade, global presence, export council
National Education Policy (NEP) 2020	National policy document	https://www.education.gov.in/sites/upload_files/mhrd/files/NEP_Final_English_0.pdf	Education reforms and digital pedagogy for Ayush

## 3 Observations

**Figure 2:**
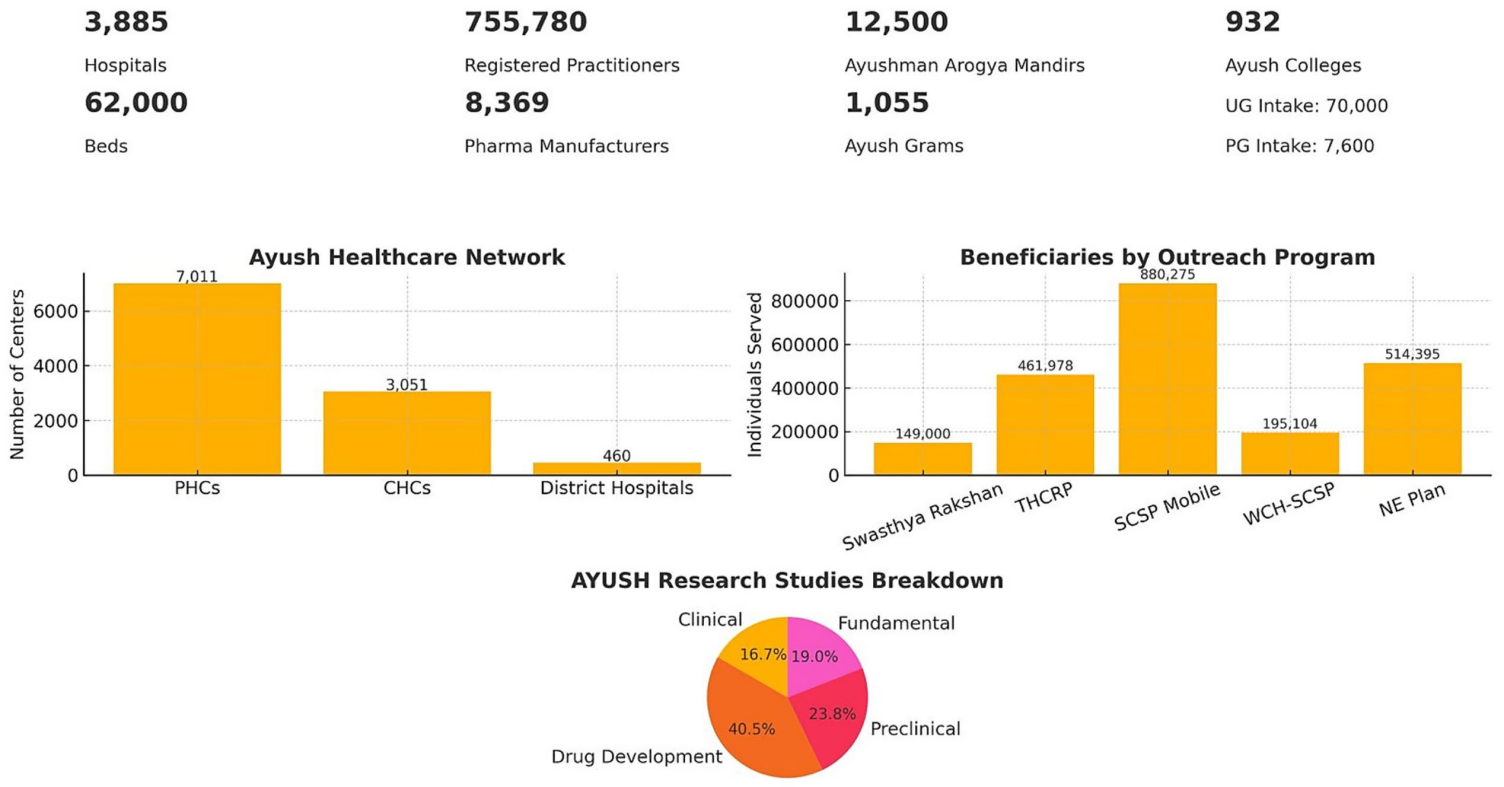
Overview of the AYUSH healthcare landscape in India. The infographic summarizes key statistics including the number of hospitals, beds, registered practitioners, pharmaceutical manufacturers, Ayushman Arogya Mandirs, Ayush Grams, and AYUSH colleges with their intake capacity. Bar graphs depict the AYUSH healthcare network (Primary Health Centres, Community Health Centres, and District Hospitals) and beneficiaries served through major outreach programs (Swasthya Rakshan, THCRP, SCSP Mobile, WCH-SCSP, and NE Plan). A pie chart illustrates the distribution of AYUSH research studies across drug development, preclinical, clinical, and fundamental categories.

Observations for this policy review were derived from both primary and secondary data sources, enabling a comprehensive analysis. The findings are organized under six thematic subheadings: policy framework, infrastructure and manpower, public health initiatives and outreach, digital integration, and global outreach. The cumulative data from major sections is summarized in Figure 2.

### 3.1 Policy framework for Ayush integration into PHC (primary health care)

#### 3.1.1 Healthcare

India’s commitment to mainstreaming Ayush into public health has been guided by evolving national policies. The National Health Policy (NHP) 2002 initiated efforts to incorporate Indian Systems of Medicine into health delivery, emphasizing access, affordability, and pluralism. The updated NHP 2017 advanced this vision by explicitly endorsing medical pluralism—defined as the coexistence and integration of multiple systems of healthcare—and by proposing structured, evidence-based inclusion of Ayush into Primary Health Care (PHC) (4, 5). The 2017 policy outlines a three-dimensional strategy for Ayush mainstreaming (6).

1. Service delivery through co-location in PHCs (Primary Health Centres) and CHCs (Community Health Centres), the backbone of India’s rural health infrastructure.
2. Research and evidence generation, particularly for prevention and chronic care management.
3. Education and human resource development

Complementing this policy vision, India’s leadership during its G20 presidency in 2023 culminated in the Gujarat Declaration, a high-level strategic document calling for the global integration of traditional medicine into PHC systems. This further enhanced India’s visibility in international health policy discussions (7). Hence for effective implementation efforts are being made through capacity building, quality assurance, and stakeholder collaboration across sectors (8).

In addition, the National List of Essential Medicines (NLEM), which guides the availability of key drugs in public health facilities and has traditionally focused on conventional (allopathic) medicines (9) has been recently updated now including Ayush formulations. Additionally, the Ministry of Ayush has created system-specific Lists of Essential Medicines (LEMs) for Ayurveda, Unani, Siddha, and Homoeopathy. These lists define which classical formulations should be prioritized for procurement and rational use in government-run Ayush hospitals (10).

Ayush services are now covered under India’s national health insurance scheme (PM-JAY) through 172 treatment packages offered by 27 insurers. In addition to insurance inclusion, the Ministry of Ayush is strengthening integration through policy reforms, sensitization of stakeholders, cross-referral pathways, digital health systems, and clinical protocol harmonization. These measures aim to enable accountable, interoperable healthcare while ongoing discussions with Insurance Regulation Authority of India (IRDAI) seek to incorporate Ayush more comprehensively under India’s ₹5 lakh (USD 5,850) family health coverage, promoting a holistic and inclusive care model (11–13).

#### 3.1.2 Education and research

Significant governance reforms have taken place in Ayush education and regulation. The older regulatory bodies—the Central Council of Indian Medicine (CCIM) and the Central Council for Homoeopathy—were replaced in 2020 by:

- NCISM: National Commission for Indian System of Medicine
- NCH: National Commission for Homoeopathy

The NCISM and NCH regulate curricula and enforce ethical standards across the country. With NEP 2020, Ayush education now incorporates Indian Knowledge Systems (IKS), digital tools, interdisciplinary learning, and international fellowships. The institutions also offer doctoral and international fellowship programs in coordination with International Council for cultural Relations (ICCR) and Ministry of External Affairs, hosting students from 32 countries.

Policy reforms in Ayush research have focused on enhancing scientific rigor and ethical integrity through standardized guidelines and regulatory frameworks. Research must comply with OECD principles for preclinical studies, CTRI registration and IEC approval for clinical trials, and follow reporting standards like ARRIVE, STROBE, PRISMA, and CONSORT. Additionally, the ICMR’s Ethical Guidelines and its specific addendum for Ayush research provide a robust framework to ensure ethical conduct and quality in traditional medicine research.

Together, these institutions enable Ayush systems to not only expand service delivery but also generate evidence needed for mainstream health policy and international credibility.

#### 3.1.3 Quality control and standardization

On the pharmaceutical side, in addition to GMP certification given to Ayush pharma manufacturing companies, the Pharmacopoeia Commission for Indian Medicine and Homoeopathy (PCIM&H) has been established as the apex body to develop quality standards for drugs. The One Herb, One Standard initiative—launched jointly with the Indian Pharmacopoeia Commission—aims to harmonize the standards used for identifying and testing medicinal plants across systems and agencies. Drug safety is regulated under the Drugs and Cosmetics Act, ensuring centralized oversight. India is also an active participant in the WHO’s International Regulatory Cooperation for Herbal Medicines (IRCH) and aligns with WHO Good Manufacturing Practices (GMP) to strengthen quality assurance and global credibility of its traditional medicine products. On the services part National Accreditation Board for Hospitals and Healthcare providers (NABH) for hospitals and National Assessment and Accreditation Council (NAAC) for educational institutions are implemented on par with Standards.

These policy, institutional, and regulatory mechanisms create an enabling ecosystem for Ayush to function as a co-equal component of India’s public health system. They also align with international goals of promoting culturally sensitive, inclusive, and evidence-informed healthcare models.

### 3.2 Sectoral infrastructure and workforce

In order to implement the policies made the next immediate requirement is infrastructure and Ayush Workforce.

#### 3.2.1 Healthcare

As of 2024, India’s Ayush public health infrastructure includes 3,885 hospitals with over 62,000 beds and more than 755,780 registered Ayush practitioners. Ayush services have been co-located in 460 District Hospitals, 3,051 Community Health Centres (CHCs), 7,011 Primary Health Centres (PHCs), and over 3,000 additional health facilities under the National Health Mission (NHM). This widespread deployment underscores the increasing institutionalization of traditional medicine in India’s Primary Health Care (PHC) system (14).

Under the National Ayush Mission (NAM), over 12,500 Ayush Health and Wellness Centres (AHWCs) have been established. These centers provide preventive, promotive, and curative care, including yoga sessions, consultations, non-communicable disease (NCD) screenings, and dietary counselling. These centers known as Ayushman Arogya Mandirs serve as flagship AHWCs.

The Directorate General of Health Services (DGHS) now hosts a dedicated Ayush vertical. Indian Public Health Standards (IPHS) guidelines have been updated to include Ayush. Essential medicine lists have been issued, that allow to stock Ayush medicines at Jan Aushadhi Kendras (Public medicine stores). A separate Ayush sub-sector skill council is established for training of existing HR by upskilling, new skilling and re skilling programs.

#### 3.2.2 Education and research

India’s Ayush education ecosystem includes 932 colleges and an annual intake of nearly 70,000 undergraduates and 7,600 postgraduate students. All India NEET and PG entrance examinations for traditional medicine, and skill development courses specifically targeting paramedical staff such as traditional medicine therapists and nurses have been introduced.

In parallel, India has built a substantial institutional research ecosystem. The Institute of Research and Training in Ayurveda (ITRA), Jamnagar an Institute of National Imminence, the All India Institute of Ayurveda (AIIA) in New Delhi and Goa, and the National Institute of Ayurveda, Jaipur as well as some other institutions under the Ministry of Ayush serve as the apex institutions for advanced education, clinical care, and integrative research in Ayurveda. Similarly, Institutes like National Institute of Homeopathy, Narela, National Institute of Siddha, Chennai serve as apex institutes of Homeopathy and Siddha traditional medicines. Additionally, Research council ecosystem has a top down model reaching the most remote areas. These centers are regionally distributed to study diverse health needs and traditional practices, and contribute significantly to the documentation, validation, and application of classical and local health traditions. Similarly, for all traditional medicine branches, the research councils work through their peripheral institutes and several other regional institutes established all over the country.

#### 3.2.3 Quality control and standardization

The Ayush sector is progressively building its human resource capacity in the areas of quality control and standardization to ensure the safety, efficacy, and credibility of traditional medicine. Currently, India has over 8,369 licensed ASU&H drug manufacturing units, supported by 29 State Drug Testing Laboratories and several central and private labs approved under the Drugs & Cosmetics Act. However, the workforce dedicated to regulatory and quality assurance roles remains limited, with small number of Ayush drug inspectors.

To address this, the Ministry of Ayush and affiliated institutions like the Pharmacopoeial Commission for Indian Medicine & Homoeopathy (PCIM&H) offer specialized training programs. These include the *Certificate Course in Quality Control of ASU&H Drugs*, along with modules on GMP, phytochemical analysis, and drug testing protocols. Such capacity-building initiatives aim to equip technical personnel—pharmacists, analysts, and regulatory staff—with the necessary skills to uphold high standards in Ayush drug manufacturing and ensure compliance with national and international quality norms. There are more than 300 NABH accredited Ayush facilities in India as of 2025.

### 3.3 Public health outreach and awareness

As of the most recent National Sample Survey Office (NSSO) survey (2022–2023), awareness of Ayush systems stands at 94–96%, with usage rates exceeding 50% nationally (52.9% urban, 46.3% rural) (15–17). Which clearly demonstrates the position of Ayush systems in India. The outreach can be broadly understood in various contexts as following:

#### 3.3.1 Integrating traditional medicine into primary health care in India

India’s integration of Ayush into Primary Health Care (PHC) is driven by a global and national recognition of the value of holistic, person-centered healthcare. This model, which emphasizes physical, mental, emotional, and spiritual well-being, is increasingly aligned with the principles of general practice and community-based healthcare. Ayush systems are not only deeply rooted in India’s cultural history but have now been systematically integrated into the country’s public health infrastructure. This integration enhances healthcare access, especially in rural and tribal areas where traditional medicine is often the first point of care (18, 19).

Several initiatives have been undertaken by various ministries, institutes, and organizations in India to strengthen the integration of Ayush into primary healthcare. The Ministry of Ayush has launched numerous programs, under the initiative called NAM and the integration of Ayush services into the National Programs (7). Notably, the Central Council for Research in Siddha (CCRS) has successfully deployed a traditional Siddha formulation, Nilavembu Kudineer for the prevention and management of dengue and chikungunya. Yoga has also been promoted through initiatives like the establishment of Preventive Health Care Units by the Morarji Desai National Institute of Yoga (MDNIY) in CGHS wellness centers across Delhi and National Capital Region. The Ministry of Defence has also established Ayurveda OPDs in Armed Forces Medical Services hospitals and Cantonment Board hospitals. These efforts collectively signify the government’s commitment to fostering an inclusive, multi-faceted healthcare system that marries traditional practices with contemporary healthcare delivery (20).

#### 3.3.2 Flagship national health integration programs

The initiative NAM, launched in 2014, has facilitated a network of Integrated Ayush Hospitals, dispensaries, and co-located services across 7,011 PHCs, 3,051 CHCs, and 460 District Hospitals. Community outreach has extended to over 1,055 villages through the Ayush Grams initiative. The Ayush health and wellness centers called Ayushman Arogya Mandirs, 12,500 in number—serve as Ayush-led primary care centers delivering yoga, NCD screening, dietary counselling, and health education. These centers are distinct from allopathic facilities and emphasize preventive, promotive, and participatory healthcare.

Ayush interventions have been integrated into national health programs such as the NPCDCS, where over 1.89 million patients were screened and 175,000 enrolled in three pilot districts. Programs like the National Program for Musculoskeletal Disorders, Supraja (a program for maternal health), Vayo Mitra (a program for geriatric care), Ayurvidya (introduced for school health), and mobile medical units (MMUs) further demonstrate wide adoption (9).

#### 3.3.3 Integration into other national programs

The Ministry of Ayush, has executed several outreach programs aimed at enhancing access to Ayush-based healthcare among underserved populations. While these efforts represent significant progress, they remain focused on targeted regions and subsets of the total eligible populations, highlighting the need for continued scale-up and systematic evaluation (21).

- Swasthya Rakshan Programme: A health promotion and disease prevention initiative implemented in selected regions to spread awareness about Ayush-based preventive care. It served over 149,000 individuals with services including lifestyle counseling, seasonal health advisories, and local herbal remedy promotion.
- Tribal Health Care Research Programme (THCRP): This program addresses the health needs of indigenous (tribal) communities. It has reached over 461,978 individuals and documented 878 Local Health Traditions (LHTs), showcasing India’s vast ethnomedical knowledge systems. However, with India’s tribal population exceeding 104 million, further expansion remains a priority.
- Scheduled Caste Sub Plan (SCSP) – Mobile Health Care Program: Provides mobile Ayush healthcare services to Scheduled Caste communities, serving 880,275 individuals. These programs are tailored to promote equity in healthcare delivery among historically disadvantaged groups.
- Women and Child Health (WCH) under SCSP: Targets maternal and child health within Scheduled Caste populations, having served 195,104 patients. The initiative includes antenatal care, child nutrition, and Ayurvedic postpartum therapies.
- Ayush Centres under North East Plan: Established in remote and geographically challenging regions of Northeast India, 19 centers have provided primary Ayush healthcare to over 514,395 people. These centers serve as models for integrative healthcare in difficult-to-reach areas.

In addition, select components of the Reproductive and Child Health (RCH) program have incorporated Ayush systems with promising results. The Central Council for Research in Ayurvedic Sciences (CCRAS) piloted the integration of Ayurvedic antenatal care protocols—particularly a classical pregnancy care regimen (Garbhini Paricharya)—at the primary health centre (PHC) level in regions like Gadchiroli, Maharashtra and Himachal Pradesh, yielding encouraging results in maternal health. These efforts reflect a growing institutional commitment to evidence-informed integrative healthcare.

Furthermore, CCRAS and other Ayush research councils operate peripheral institutes across India that combine structured outreach, clinical services, and health systems research. Many of these centers offer outpatient (OPD) services, and select ones also run inpatient (IPD) units, providing accessible Ayush-based care. While some are disease-specific (e.g., for cardiovascular diseases in New Delhi), others focus on therapeutic modalities (e.g., Panchakarma in Kerala) or broader thematic areas. This dual role enhances both research capacity and access to traditional medicine within the primary care ecosystem.

CCRAS has also collaborated with tertiary care institutions like Safdarjung Hospital in New Delhi to build integrative models for managing conditions such as osteoarthritis. At the national level, the Ayush-ICMR Advanced Centre for Integrative Health Research (AI-ACIHR) at AIIMS aims to build rigorous scientific foundations for such integrative care practices under the Indian Council of Medical Research (ICMR) umbrella. Together, these initiatives reflect India’s strategic emphasis on scalable, evidence-based integration of traditional systems into modern public health frameworks (22).

#### 3.3.4 Malnutrition free India (Kuposhan Mukt Bharat) and Ayush-based nutritional strategies

Ayush plays an active role in India’s public health nutrition efforts through its engagement with the “Kuposhan Mukt Bharat” (Malnutrition-Free India) initiative. The Ministry of Ayush has released dietary advisories that align traditional Ayurvedic nutritional principles with contemporary public health standards. These guidelines are seasonally and regionally adapted and offer age- and condition-specific dietary recommendations for children, pregnant and lactating women, and individuals with anemia and undernutrition (23).

To reinforce community-level implementation, four targeted yoga modules—designed for children (ages 3–6), adolescent girls, pregnant women, and lactating mothers—were developed and disseminated through the Integrated Child Development Services (ICDS) platform via Anganwadi (community health) workers. These modules were jointly supported by the Ministries of Ayush and Women and Child Development under the broader scheme of “Suposhit Bharat” (Well nourished India) through well balanced Ayurveda diet (24).

The Ministry of Ayush also contributed traditional knowledge inputs to the revision of nutrition guidelines under the National Food Security Act (NFSA), 2013 (23). Ayurveda-inspired dietary counselling is a key service at Ayush Health and Wellness Centres (HWCs) established under NAM.

Additionally, research and field-level applications are underway to explore Rasayana-based dietary formulations and Ayurveda-inspired recipes for managing nutritional deficiencies such as childhood malnutrition and anemia (25). These initiatives emphasize preventive, promotive, and culturally contextual approaches to nutrition-sensitive healthcare.

Complementary efforts include the establishment of *Poshan Vatikas* (gardens with nutritional fruit bearing plants) and Ayurveda Nutrigardens in schools and communities, integration of Ayurveda-based dietary education in Ekalavya Model Residential Schools through collaborations with the Ministry of Tribal Affairs (MoTA) and the Ministry of Women and Child Development (WCD), and dissemination of Ayurveda *Aahar* (Ayurveda based diet) guidelines to promote sustainable, locally sourced, and culturally relevant nutrition practices.

These programs are executed jointly by Ayush research councils, state health departments, and local health workers. Through these, cost effective Ayush products and services are made available to everyone’s doorstep.

India’s model of Ayush integration in PHC serves as a replicable framework for culturally contextual, affordable, and scalable traditional medicine deployment worldwide. With its deep institutional foundations, cross-sectoral engagement, and global vision, the Ayush ecosystem is poised to become a cornerstone of both national health planning and global public health innovation.

These descriptions have been simplified into a flowchart illustrating how Traditional Medicine can be integrated across PHC levels (Figure 1).

### 3.4 Digital integration and information systems

The Ayush Grid (26)—a comprehensive digital health initiative—includes platforms such as A-HMIS (Ayush Hospital Management Information System), e-Aushadhi (digital supply chain), NAMASTE Portal (terminology and morbidity reporting), e-Charak (herbal networks), and Tools like eSanjeevani allow remote Ayush consultations.

Research tools such as the Ayush Research Portal, Traditional Knowledge Database Library (TKDL), Research Management Information System (RMIS), and e-Grantha (library automation and networking) facilitate transparency and access to clinical and traditional knowledge repositories.

Pharmacovigilance and adverse drug reaction (ADR) reporting are supported through tools like Ayush Suraksha portal and Suraksha Ayush application. Further to these advancements, interoperability with mainstream health IT systems under the Ayushman Bharat Digital Mission (ABDM) has started evolving.

A recent WHO technical brief, *Mapping the Application of Artificial Intelligence in Traditional Medicine*, developed with input from Ministry of Ayush, highlighted the potential of AI to enhance traditional medicine practices while underscoring ethical considerations such as data bias, patient privacy, informed consent, and safeguarding cultural heritage, reinforcing the importance of responsible digital integration (27).

### 3.5 Global positioning and collaborations

Traditional medicine systems are gaining renewed global relevance as countries seek to build more culturally appropriate, preventive, and cost-effective health systems. According to the WHO Global Report on Traditional and Complementary Medicine (TCM) 2019, 88% of WHO Member States (170 countries) report using some form of Traditional, Complementary and Integrative Medicine (TCIM), with 107 countries having national offices and 75 maintaining dedicated research institutes (25). The WHO Traditional Medicine Strategy 2025–2034 further emphasizes the importance of integrating TCIM into national health systems in an evidence-based, safe, and equitable manner.

At the global market level, the demand for herbal medicines—a core component of many TCIM systems—is projected to grow from USD 233 billion in 2024 to over USD 437 billion by 2032 (28). In many developed countries, use of TCIM is substantial: an estimated 24–71% of Europeans in different countries and 40% of US adults report having used some form of TCIM in recent years (29, 30).

India hosts the WHO Global Centre for Traditional Medicine (GCTM) in Jamnagar. International campaigns such as “Heal in India” and Ayush visas for Medical Value Travel (MVT) enhance India’s soft power. India has 42 Ayush Information Cells across 38 countries and 15 academic Chairs at global universities (31).

Formal recognition of Ayurveda systems exists in Sri Lanka, UAE, Oman, Malaysia, Nepal, Colombia, Tanzania, Hungary, Serbia, and others. Siddha is recognized in Sri Lanka and Malaysia; Sowa Rigpa in Bhutan and Mongolia. These recognitions result from diplomatic efforts by the Ministry of Ayush (32). Also, some Ayush practices, training courses and some forms of practice have been recognized in various countries across the globe like Switzerland, Australia, etc. (33).

Academic Collaboration and Trade Promotion: India has launched the Ayush Export Promotion Council (Ayushexcil) to boost global trade, regulatory cooperation, and academic partnerships (34). In fiscal year 2023–24, India’s exports of Ayush and herbal products grew by 3.6%, rising from USD 628.54 million to USD 651.17 million within a year (35). A dedicated IC scheme provides support to drug manufacturers/service providers to boost export.

Despite these significant strides in global outreach and recognition, strategic efforts are still needed to position India as the global leader in traditional medicine. Strengthening regulatory harmonization, evidence-based validation, and international collaborations will be key to realizing this potential.

## 4 Strategic future directions for global integration of Ayush

Looking forward, India’s traditional medicine sector can play a pivotal role in shaping the future of integrative healthcare — both domestically and globally. Several strategic directions can guide this evolution:

- Digital Education Ecosystem: Establish dedicated centers for Ayush educational technology and innovation, including simulation-based Panchakarma labs, virtual learning platforms, and AI-driven pedagogy. Integration with NEP 2020 will ensure global competitiveness and skill-based training (36).
- Policy-Oriented Implementation Science: Develop collaborations between Ayush institutions and public health bodies to evaluate the scalability, fidelity, and cost-effectiveness of Ayush Health & Wellness Centres under the National Ayush Mission. This will help generate implementation evidence to inform health policy and financing.
- Global Harmonization of Quality Standards: Work closely with WHO, ISO, and partner countries to harmonize protocols, safety standards, and pharmacopoeial specifications. This is essential for enabling cross-border regulatory acceptance and scaling integrative care models (37).
- Artificial Intelligence in Clinical Decision Support: Leverage AI tools trained on Ayush clinical data to enhance diagnosis, patient stratification, and treatment personalization — especially in the domains of non-communicable diseases and mental health.
- Transdisciplinary Medical Education: Embed comparative modules on Ayush and biomedicine into MBBS, BAMS, and allied health curricula. This supports NEP 2020’s call for transdisciplinary and integrative education and promotes mutual understanding among systems.
- One Health Integration via Ayush-Biodiversity Interface: Position Ayush systems within One Health frameworks, particularly for addressing zoonotic disease prevention, antimicrobial resistance (AMR), and climate-sensitive health challenges through sustainable, plant-based solutions.

All the strategic plans require a strong evidence informed research backup therefore Research methodologies become and integral part of Primary Healthcare Integration.

## 5 Research perspectives and methodologies in traditional medicine (TM) within primary healthcare

India’s primary healthcare system, particularly in rural and semi-urban areas, encounters diverse challenges that conventional biomedical approaches alone cannot fully address. Integrating Traditional Medicine (TM)—including codified systems like Ayurveda, Yoga, Unani, Siddha, and Homeopathy, along with local ethnomedical practices—has opened avenues for inclusive and culturally grounded healthcare delivery, offering new research frontiers.

### 5.1 Ethnomedical documentation and preservation

Community-based ethnographic studies have helped document region-specific health practices and therapies. Programs like the Tribal Health Care Research Programme (THCRP) have recorded 878 Local Health Traditions, while the Medico-Ethno Botanical Survey (MEBS) led by CCRAS collects and validates folklore-based health claims (38, 39). Although concerns persist about the marginalization of non-codified systems, including tribal medicine (40, 41), they are being addressed through safeguards focused on preserving indigenous knowledge and ensuring equitable benefit-sharing.

### 5.2 Public health, phytopharmacology, and clinical validation

TM research increasingly addresses lifestyle disorders, immunity, and preventive care—especially post-COVID-19. Phytopharmacological studies led by Council of Scientific and Industrial Research (CSIR) and collaborative missions with Ayush have advanced herbal drug development and standardization. Successful examples include development of Ayush-64 for malaria, which was later repurposed for COVID-19, and validation of classical formulations like Ashwagandha (*Withania somnifera*), Guduchi (Tinospora cordifolia), and Pippali (*Piper longum*) in scientifically designed studies (42). Examples of promising research outputs include clinical studies on Terminalia arjuna in cardiovascular diseases (43, 44). Guduchi (*Tinospora cordifolia*) for immunomodulation (45), and Ashwagandha (*Withania somnifera*) for stress reduction and mental well-being (46, 47). However, there remains a need for larger, multi-center trials to validate such findings at scale.

Randomized controlled trials (RCTs), though still limited in number, have evaluated TM therapies in conditions like psoriasis and osteoarthritis. Pragmatic and adaptive trials are gaining favor, given the multi-component nature of TM. Pragmatic controlled trials like the German RCT comparing Ayurvedic treatments with conventional care, and longitudinal studies at SDM Ayurveda Hospital, exemplify context-sensitive trial models (48, 49).

### 5.3 Personalized and genomic research

Ayurvedic concepts such as Prakriti (constitutional types) are now being explored through modern genomic research. Studies in Ayurgenomics are validating traditional classifications by linking them to molecular and genetic markers, enabling a new paradigm of personalized healthcare aligned with behavioral and metabolic traits (50–52).

### 5.4 Community participation and digital innovation

Community-Based Participatory Research (CBPR) models help ensure ethical and culturally appropriate research. By involving local stakeholders, these approaches promote trust and sustainability. Concurrently, digital health tools, telemedicine, AI-driven analysis, and mobile applications are improving monitoring, accessibility, and pharmacovigilance in TM research.

Despite substantial progress in the outreach and recognition, critical gaps remain that warrant systematic evaluation and strategic planning—paving the way for informed integration of Ayush into primary healthcare systems, as discussed in the following section.

## 6 Critical reflections on integrating Ayush into primary healthcare

India has made remarkable progress in integrating Ayush into its primary healthcare system, supported by strong policy frameworks and robust institutional mechanisms. Diverse models across states, expanding cross-referral pathways, and a steadily growing workforce reflect this momentum. The Ayush Research Portal now houses over 43,000 studies, spanning clinical research (~7,000), drug development (~17,000), preclinical research (~10,000), and fundamental studies (~8,000), offering a rich evidence base to inform clinical guidelines, academic curricula, and frontline practice. Looking ahead, emphasis on developing customized R&D protocols for Traditional Medicine, global harmonization of pharmacopoeial standards, innovative supply chain models for improved medicine accessibility, and expanded cultivation of medicinal plants will further strengthen integration. Conducive IPR policies that promote research while safeguarding the rights of knowledge holders will be crucial. Promising initiatives such as ICD-TM2 implementation through the Ayush Health Information Management System (AHIMS), responsible integration of AI in TM research and practice, advanced “Ayurveda Biology” projects including metabolomics studies, and personalized predictive–preventive healthcare models are emerging frontiers. How Ayush shapes and leads these developments will be pivotal to building a sustainable, globally relevant, and inclusive healthcare ecosystem.

## 7 Future possibilities for traditional medicine in primary health care interventions

Traditional Medicine (TM) is increasingly recognized as a valuable component of primary health care (PHC), offering cost-effective, culturally acceptable, and patient-centered approaches. The World Health Organization (WHO) has emphasized integrating TM into national health systems to address gaps in preventive, promotive, and curative care, particularly in low- and middle-income countries (53, 54). TM’s strength lies in its holistic and personalized framework, which addresses physical, mental, and social dimensions of health, making it well-suited for non-communicable disease (NCD) prevention, mental health promotion, and lifestyle modification (55, 56).

For instance, during the influenza outbreaks in early 20th century, Ayurvedic interventions such as fumigation therapy (Dhoopana) and the oral administration of herbal decoctions were widely adopted. These practices not only supported individual immunity but also contributed to community-level disease prevention and health promotion (57). During the COVID-19 pandemic, Ayush-based preventive and supportive strategies were widely implemented. These included usage of prophylactic formulations like herbal decoctions (Ayush Kadha), immunomodulatory formulations and medicinal oils for nasal therapy. A prominent example was the AYURAKSHA kit—developed by the All India Institute of Ayurveda, Delhi—which reported reduced COVID-19 incidence among Delhi Police personnel in a non-randomized trial (58). Similarly, systems such as Siddha and Unani contributed region-specific prophylactic and therapeutic protocols, while Yoga and Naturopathy approaches were integrated into mental health and well-being initiatives during COVID pandemic. The pluralistic nature of Ayush thus offers diverse tools for enhancing population health, but also highlights the need for robust evaluation frameworks that respect system-specific methodology.

The COVID-19 pandemic underscored the limitations of conventional PHC in managing chronic conditions, mental health burdens, and emerging challenges such as antimicrobial resistance (AMR) (59–61). Evidence suggests that TM interventions—such as Ayurveda’s Rasayana therapy, —can complement conventional strategies in immune modulation, stress reduction, and infection control (62, 63). Additionally, TM’s emphasis on diet, daily routines, and seasonal adaptation aligns with modern preventive health paradigms.

Looking forward, TM could play a transformative role through:

1. Integrative PHC Models – Combining evidence-based TM with biomedical protocols for prevention/wellness, addressing chronic disease management including NCD and mental health care from grass-root level. TM approaches such as personalized dietary guidance, detoxification, and herbal therapies offer supportive care for NCDs, which account for 74% of global deaths annually (64). Rasayana therapies like *Triphala*, *Ashwagandha*, and *Amalaki* show neuroprotective and immunomodulatory benefits in the elderly (65–68).
2. Addressing new health care challenges (AMR Mitigation) – Promoting rational research, creating evidence in exploring utility of traditional medicine in primary health care addressing latest health care challenges using antimicrobials and immunomodulators against the growing challenge that needs to be redressed. Herbal formulations with immunomodulatory and antimicrobial potential—like *Ocimum sanctum*, *Tinospora cordifolia*, *Curcuma longa*, and *Withania somnifera*—lowers AMR risk (69).
3. Community-Based Health Promotion – Empowering TM practitioners as frontline PHC providers. With Strategic policy support, rigorous research, and capacity building will ensure safe, effective, and equitable integration into future Public health frameworks. Ayush systems offer interventions such as *Ashwagandha* for anxiety (70), *Bacopa monnieri*, *Shirodhara*, and yoga-meditation programs for cognitive well-being (71–76). Ayush systems offer interventions such as *Ashwagandha* for anxiety (73), *Bacopa monnieri*, *Shirodhara*, and yoga-meditation programs for cognitive well-being (71–76), which are testaments of possibilities.
4. Digital Health Platforms – Delivering personalized TM-based lifestyle interventions and tele consultations making the reach of traditional medicine beyond and far across rural population.

Going forward, integration of TM into PHC will require investment in implementation science, interprofessional education, and robust regulatory frameworks. Strategic alignment with WHO and global policy directions will enhance TM’s contributions to an equitable, person-centered healthcare future.

## 8 Conclusion

**Figure 3:**
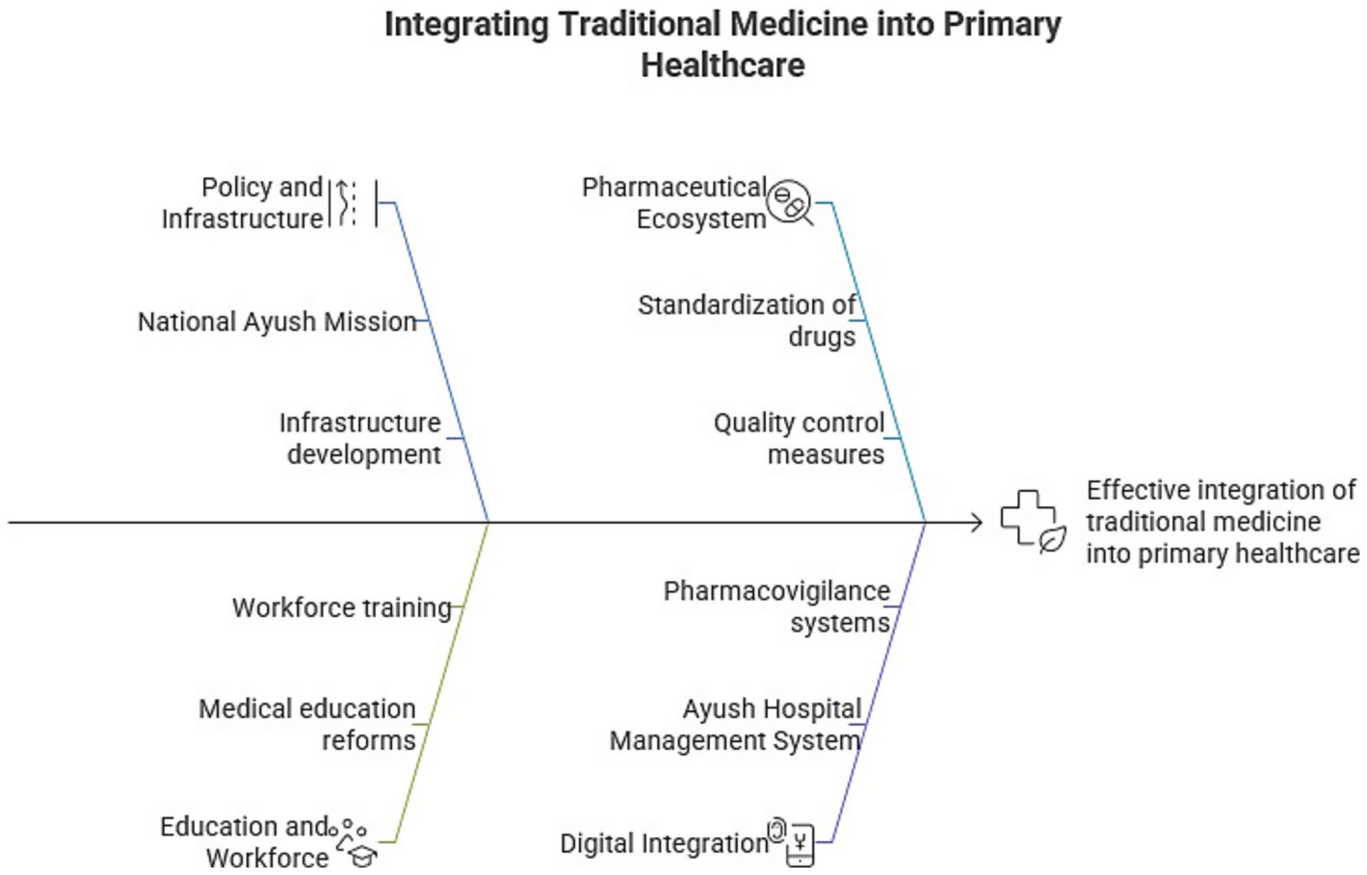
Flowchart illustrating key components essential for integrating Traditional Medicine into primary healthcare, including policy support, infrastructure, education, pharmaceutical standardization, digital systems, and pharmacovigilance mechanisms (Generated with the assistance of Napkin AI).

India’s efforts to integrate Ayush systems into primary healthcare reflect a broader attempt to balance tradition with innovation in public health delivery. While significant progress has been made through service expansion, research, policy support, and global partnerships, active efforts are being implemented for overcoming challenges for ensuring consistent quality, workforce capacity, and evidence-based practice. The growing body of research, digital innovation, and community outreach are promising trends, thus are being met with rigorous evaluation and adaptive implementation models. The comprehensive roadmap for integrating traditional medicine into primary healthcare, along with the key implementation strategies, is summarized in Figure 3.

A balanced path forward would involve sustained investment in health systems research, capacity building, and participatory governance to ensure Ayush systems complement rather than compete with existing services. With equitable integration, grounded in public health priorities and responsive to community needs, Ayush can meaningfully contribute to India’s goals for universal health coverage and to the global discourse on integrative, person-centered care.
